# Cobra Venom Factor-induced complement depletion protects against lung ischemia reperfusion injury through alleviating blood-air barrier damage

**DOI:** 10.1038/s41598-018-28724-z

**Published:** 2018-07-09

**Authors:** Chang Haihua, Wang Wei, Huang Kun, Liao Yuanli, Lin Fei

**Affiliations:** 1 Department of Emergency, the First Affiliated Hospital of Guangxi Medical University, Nanning, China; 2 Department of Anesthesiology, Affiliated Tumor Hospital of Guangxi Medical University, Nanning, China

## Abstract

The purpose of this study was to study whether complement depletion induced by pretreatment with Cobra Venom Factor (CVF) could protect against lung ischemia reperfusion injury (LIRI) in a rat model and explore its molecular mechanisms. Adult Sprague-Dawley rats were randomly assigned to five groups (n = 6): Control group, Sham-operated group, I/R group, CVF group, I/R + CVF group. CVF (50 μg/kg) was injected through the tail vein 24 h before anesthesia. Lung ischemia reperfusion (I/R) was induced by clamping the left hilus pulmonis for 60 minutes followed by 4 hours of reperfusion. Measurement of complement activity, pathohistological lung injury score, inflammatory mediators, pulmonary permeability, pulmonary edema, integrity of tight junction and blood-air barrier were performed. The results showed that pretreatment with CVF significantly reduced complement activity in plasma and BALF. Evaluation in histomorphology showed that complement depletion induced by CVF significantly alleviated the damage of lung tissues and inhibited inflammatory response in lung tissues and BALF. Furthermore, CVF pretreatment had the function of ameliorating pulmonary permeability and preserving integrity of tight junctions in IR condition. In conclusion, our results indicated that complement depletion induced by CVF could inhibit I/R-induced inflammatory response and alleviate lung I/R injury. The mechanisms of its protective effects might be ameliorated blood-air barrier damage.

## Introduction

Ischemia-reperfusion (I/R) injury is a serious clinical event which can take place in diverse conditions, such as organ transplantation, shock, cardiac arrest, thoracic surgery and multiple trauma^1,2^. Owing to the unique dual blood supply systems and physiological features, the lung is vulnerable to I/R injury^3,4^. The pathogenesis of lung ischemia reperfusion injury (LIRI) is complex and remains unclear. Therefore, more study is needed to elucidate the pathological mechanisms of LIRI and explore effective treatment methods.

Some studies confirm that lung I/R could induce sterile inflammatory eruption by activating the innate immune response^5–7^. The immune inflammatory reaction during lung I/R initiates by attracting inflammatory cells, such as lymphocytes, neutrophils, macrophages and releasing pro-inflammatory factors^8^. The complement system plays a pivotal role in activating the innate immune system. However, excessive complement activation and immune activation could lead to severe tissue injury^9,10^. Therefore, the uncontrollable complement activation is considered to be responsible for the pathogenesis mechanism of acute lung injury (ALI) induced by I/R. Inhibition of excessive complement activation might be a possible way to alleviate the tissue injury caused by I/R^11,12^.

Cobra Venom Factor (CVF) is a non-toxic protein purified from cobra venom. It is a structural analogue of complement component C3 and has the function of complement activation^13^. Treatment with CVF could deplete complement protein and achieve the desired anti-complement effect through continuously activating complement component C3 for a long time^14^. Several studies have shown that complement depletion caused by treatment of CVF plays a beneficial effect in the pathological process of transplantation^15–17^. Thus, the CVF treatment could be a possible therapeutic measure for lung I/R injury. But, as far as we know, there are few studies that have been designed to explore its effects in a LIRI animal model. This study aimed to clarify the role of complement depletion induced by CVF in blood-air barrier during LIRI development. This novel mechanism provides a potential role for complement depletion in LIRI therapeutic applications.

## Materials and Methods

### Animal Groups

Adult Sprague-Dawley rats (male, 200–250 g) provided by animal experiment center (Guangxi Medical University, China) were randomly assigned to five groups (n = 6): (1) Control group (not treated with anything), (2) Sham-operated group (underwent thoracotomy, not occluded the hilus pulmonis and reperfusion), (3) I/R (lung ischemia-reperfusion) group, (4) CVF group (only injected CVF), (5) I/R + CVF group. The animal experiment protocol was authorized and all animal experiments were carried out in accordance with the guidelines of the Animal Care and Use Ethics Committee (Guangxi Medical University, China).

### CVF Application

CVF (catalogue number: ZX0006; Shanghai Heng Fei biological technology CO. LTD, China) purified from the chinese Cobra venom, was dissolved in normal saline (Sichuan Ke Lun Pharmaceutical CO. LTD, China) and injected via the tail vein at 50 μg/kg at 24 h before anesthesia. The usage and dose of CVF were determined according to previous study^17^.

### Animal model of LIRI

The rat model of LIRI was established as we described previously^18^. Rats were anesthetized by 10% chloral hydrate (4.5 ml/kg) through intraperitoneal injection. Tracheal intubation was administrated after anesthesia. RSP1002-type small animal ventilator was applied to mechanical ventilation with breathing frequency of 80 breaths/min, tidal volume of 10 ml/kg, respiratory ratio of 1:1, inspired oxygen concentration of 100%. After underwent thoracotomy, the rats in I/R group were occluded the left hilus pulmonis (including pulmonary bronchi, artery, and vein) by a microvascular clamp for 60 minutes, followed a reperfusion of 120 minutes before closing thoracic incision. The rats in Sham-operated group had undergone thoracotomy, but were not occluded the hilus pulmonis and reperfusion. All rats were sacrificed by cervical vertebra dislocation at 2 h after operation. The left lung tissue, the venous blood, and bronchoalveolar lavage fluid (BALF) were collected. No animal mortality was recorded during the entire experiments.

### Evaluation of histological morphology

As we described previously^18,19^, rats lung tissues were fixed in 4% paraformaldehyde and embedded in paraffin. The slices (4 microns) were waxed off by hydration and xylene, stained by Hematoxylin and eosin (H&E). After conventional dehydration, transparence and sealing, stained paraffin sections were examined by light microscopy. 10 fields were examined at 200 × total magnification for each section. The damage of lung tissue was assessed with a scoring system as described previously^6^. Briefly, the first standard for evaluation was aggregation or infiltration of inflammatory cells in vessel wall or air space: 1 = only wall and air space, 2 = few cells infiltration in air space, 3 = intermediate, 4 = severe aggregation of inflammatory cells in air space. Second standard for evaluation was hyaline membrane formation and interstitial congestion: 1 = normal, 2 = moderate (>25% of lung section), 3 = intermediate (25%–50% of lung section), 4 = severe (>50% of lung section). Third standard for evaluation was hemorrhage: 0 = absent, 1 = present. Sections were prepared and examined by experienced pathologists who were blinded to the group assignment.

### Collection of BALF

BALF was isolated as we described previously^18^. Briefly, 5 ml of cold phosphate buffered saline (Dulbecco’s PBS; Gibco BRL, Grand Island, USA) were flushed to the lungs through the cannulated trachea to collect BALF.

### Determination of complement activity

CVF is a stable anti-complement protein and functionally resembles C3 purified from Cobra venom. To testify complement depletion induced by CVF pretreatment, complement activities were determined by the 50% haemolytic complement (CH50) activity in serum and the C3 level in the plasma and BALF. The CH50 is a screening assay which can test the functional capability of serum complement components. The level of CH50 in serum was determined by hemolytic assay^17^. In brief, serum samples were sensitized with goat anti-serum and incubated at 30 °C for 60 min to serial dilutions in gelatin veronal buffer (Sigma, St. Louis, MO, USA). After a centrifugation step (2500 g, 5 min), the supernatant fluid was collected and measured at 405 nm. The C3 levels in the plasma and BALF were determined by ELISA (catalogue number: ab157731; Abcam, Cambridge, MA) according to the manufacturer’s instructions. The quantity of C3 was standardized to a predetermined standard quantity derived from SD rat.

### Inflammatory Cytokines Measurements in Lung tissues and BALF

ELISA kits for measuring rat IL-1β, IL-6 and TNF-α (catalogue number: RLB00, R6000B and RTA00, respectively; R&D Systems, USA) were used to measure the contents of IL-1β, IL-6 and TNF-α in lung tissues and BALF according to the manufacturer’s instructions. The quantity of the cytokine was standardized to the protein content. MPO (myeloperoxidase) is a lysosomal protein stored and abundantly expressed in the neutrophils. Therefore, we determined MPO activity in lung tissues and BALF to evaluate the neutrophils infiltration. MPO activity was measured with chemical method according to the manufacturer’s instructions (Nanjing Jiancheng Biochemistry Co., Nanjing, China).

### Determination of wet/dry ratio

As we described previously^18,20^, wet/dry ratios of lung tissues were determined to assess the levels of pulmonary edema and congestion. After rats were sacrificed, the lower lobe tissues of left lung were immediately weighed, then the tissues were weighted again after dried at 60 °C for 24 h.

### Determination of Pulmonary Microvascular Permeability

The total protein in BALF could indicate the permeability of alveolar vascular. Total protein in BALF was measured by the bicinchoninic acid (BCA) assay according to the manufacturer’s instructions (Pierce, Rockford, USA).

### Western Blot Analysis

As we described previously^18,19^, the left lung tissues (50 ug) were homogenized in RIPA buffer (catalogue number: 89900; Thermo Scientific, USA) containing phosphatase inhibitor cocktail (catalogue number: 04906845001; Roche Applied Science, USA) and protease inhibitor cocktail (catalogue number: P2714; Sigma, USA). Homogenates were centrifuged at 4 °C (13,000 rpm, 20 min). The supernatant was absorbed as the total protein. The protein concentration was measured by BCA protein assay according to the manufacturer’s instructions (catalogue number: 23227; Pierce Biotechnology, USA). Twenty microgram proteins each lane were loaded on a polyacrylamide gel and transferred onto polyvinylidene difluoride membrane. Then the proteins in membrane were incubated with the primary antibodies at 4 °C: anti-AQP1 antibody (1:1000, catalogue number: ab168387; Abcam, USA), anti-ICAM-1 antibody (1:1000, catalogue number: ab206398; Abcam, USA), anti-ZO-1 antibody (1:1000, catalogue number: ab190085; Abcam, USA), anti-MMP2 antibody(1:1000, catalogue number: ab37150; Abcam, USA), anti-MMP9 antibody(1:1000, catalogue number: ab38898; Abcam, USA), anti-β-Actin antibody (1:5000, catalogue number: ab50591; Abcam, USA). The protein bands were developed by enhanced chemiluminescence (Pierce, USA). The intensities of AQP1, ICAM-1, ZO-1, MMP2, MMP9 proteins were normalized by those of β-Actin.

### Statistical analysis

All data are presented as the mean ± standard deviation (SD). (n = 6). Statistical analysis were performed using SigmaStat (Systat Software, CA, USA). The data were conducted by one-way analysis of variance followed by the Tukey test if the data were normally distributed or by one-way analysis of variance on ranks followed by the Tukey test if the data were not normally distributed. Differences were considered statistically significant at *P* < 0.05 based on two-tailed hypothesis testing.

## Results

### CVF induces complement depletion

**Figure 1:**
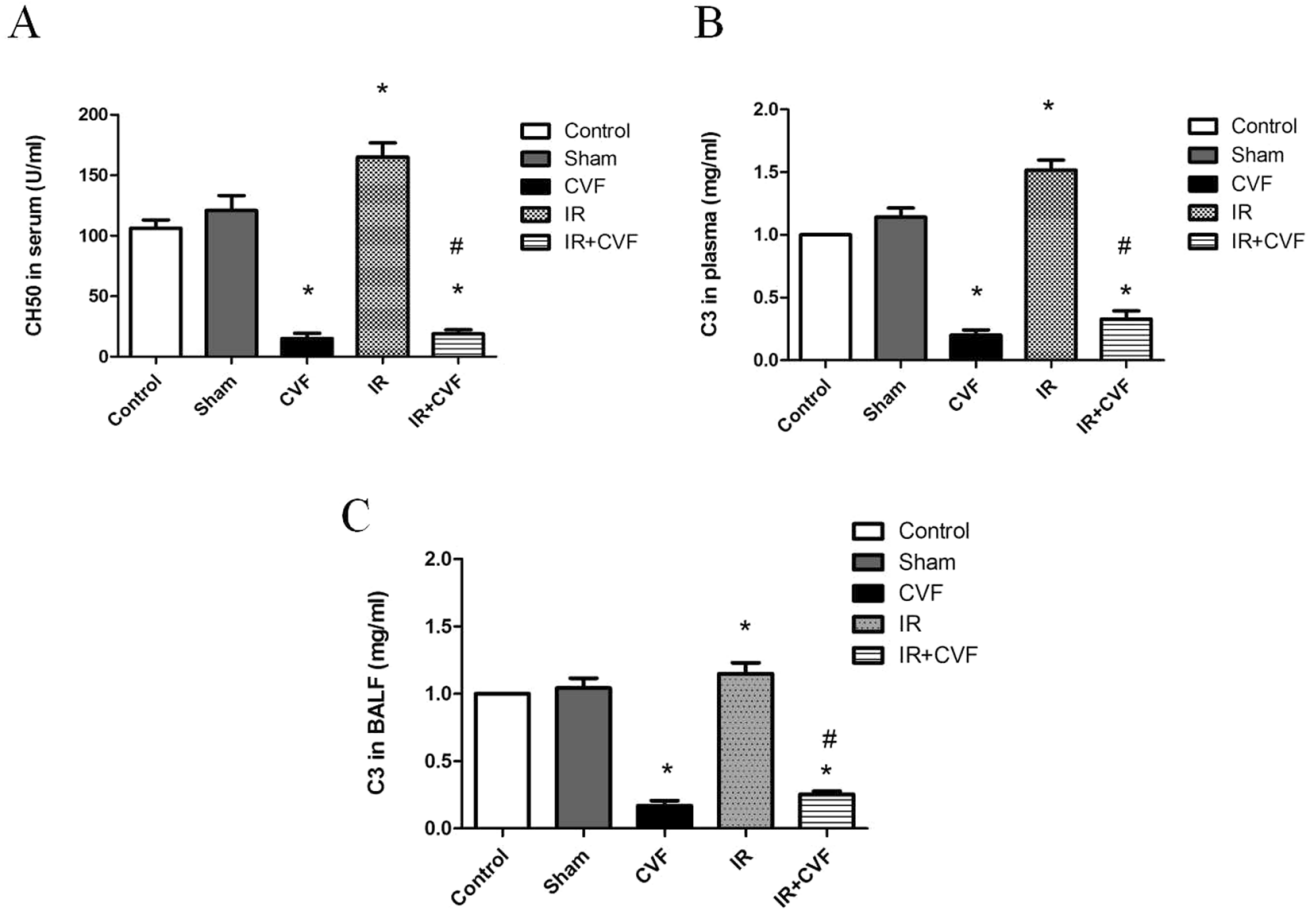
CVF decreases complement activity in plasma and BALF. (**A**) Graphic presentation of CH50 in serum. (**B**) Graphic presentation of C3 in plasma. (**C**) Graphic presentation of C3 in BALF. (^*^P < 0.05, compared with control. ^#^*P* < 0.05, compared between I/R and I/R + CVF group).

The levels of CH50 in serum and C3 in plasma and BALF were measured to assess the complement activity. Figure 1A presented the levels of CH50 in serum, Fig. 1B presented the levels of C3 in plasma, Fig. 1C presented the levels of C3 in BALF. The results demonstrated that CVF significantly reduced complement activity and could induce complement depletion in a rat model (*P* < 0.05).

### CVF pretreatment ameliorates LIRI in morphology

**Figure 2:**
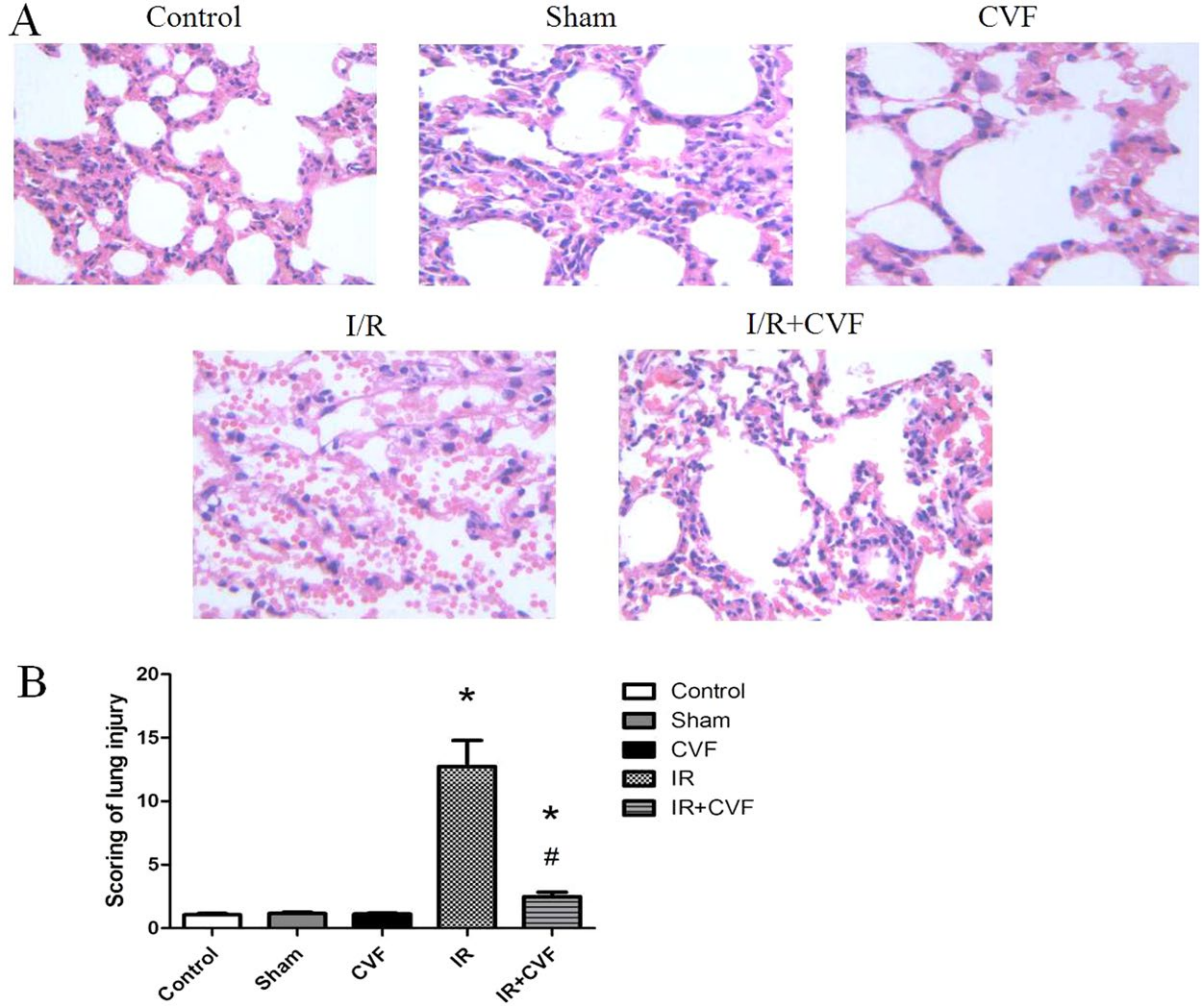
Morphological changes in Control, Sham, CVF, I/R and I/R + CVF groups by H&E staining(×200) (**A**). The scoring of lung injury shown in (**B**). (^*^P < 0.05, compared with control. ^#^*P* < 0.05, compared between I/R and I/R + CVF group).

Lung tissue staining and histology scoring were conducted to assess lung injury by I/R. Figure 2A showed the pulmonary morphology of all groups. The structures of lung tissues were intact, rarely inflammatory cells and red blood cells infiltration in control, sham and CVF groups. No difference was noted between control, Sham and CVF group (*P* > 0.05) (Fig. 2B). In I/R group, the alveolar septum had been ruptured, lung tissue structures were ambiguous, a large number of inflammatory cells and red blood cells infiltration were observed. The lung injury score was significantly higher in I/R group compared with the control group (*P* < 0.05). In I/R + CVF group, lung tissue structures were roughly intact, a small number of red blood cells and neutrophils had infiltrated into the alveolar space. Lung tissue damage was significantly alleviated in I/R + CVF group compared with I/R group (*P* < 0.05).

### CVF pretreatment inhibits inflammatory response in IR condition

**Figure 3:**
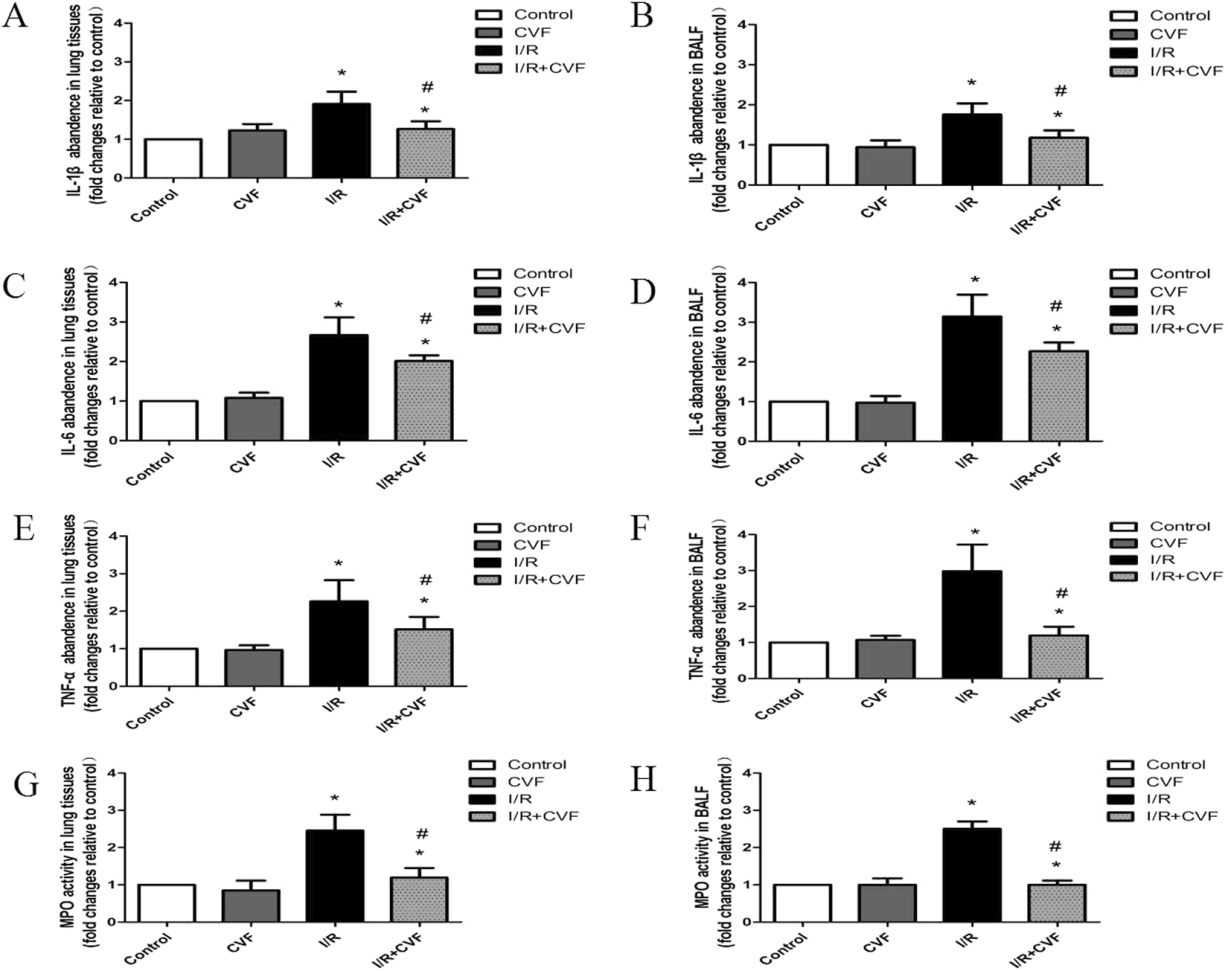
CVF pretreatment inhibits I/R-induced IL-1β, IL-6, TNF-α and MPO in lung tissues and BALF. (**A**) Graphic presentation of IL-1β abundance in lung tissues. (**B**) Graphic presentation of IL-1β abundance in BALF. (**C**) Graphic presentation of IL-6 abundance in lung tissues. (**D**) Graphic presentation of IL-6 abundance in BALF. (**E**) Graphic presentation of TNF-α abundance in lung tissues. (**F**) Graphic presentation of TNF-α abundance in BALF. (**G**) Graphic presentation of MPO activity in lung tissues. (**H**) Graphic presentation of MPO activity in BALF. (^*^P < 0.05, compared with control. ^#^*P* < 0.05, compared between I/R and I/R + CVF group).

IL-1β, IL-6, TNF-α and MPO were determinated to assess the inflammatory response in lung tissues and BALF (Fig. 3). The results showed that compared with the control group, the expression of IL-1β, IL-6, TNF-α and MPO in Lung tissues and BALF significantly increased in I/R group (*P* < 0.05). Compared with the I/R group, CVF pretreatment significantly inhibited the expression of IL-1β, IL-6, TNF-α and MPO induced by lung I/R (*P* < 0.05).

### CVF pretreatment decreases pulmonary permeability and edema in IR condition

**Figure 4:**
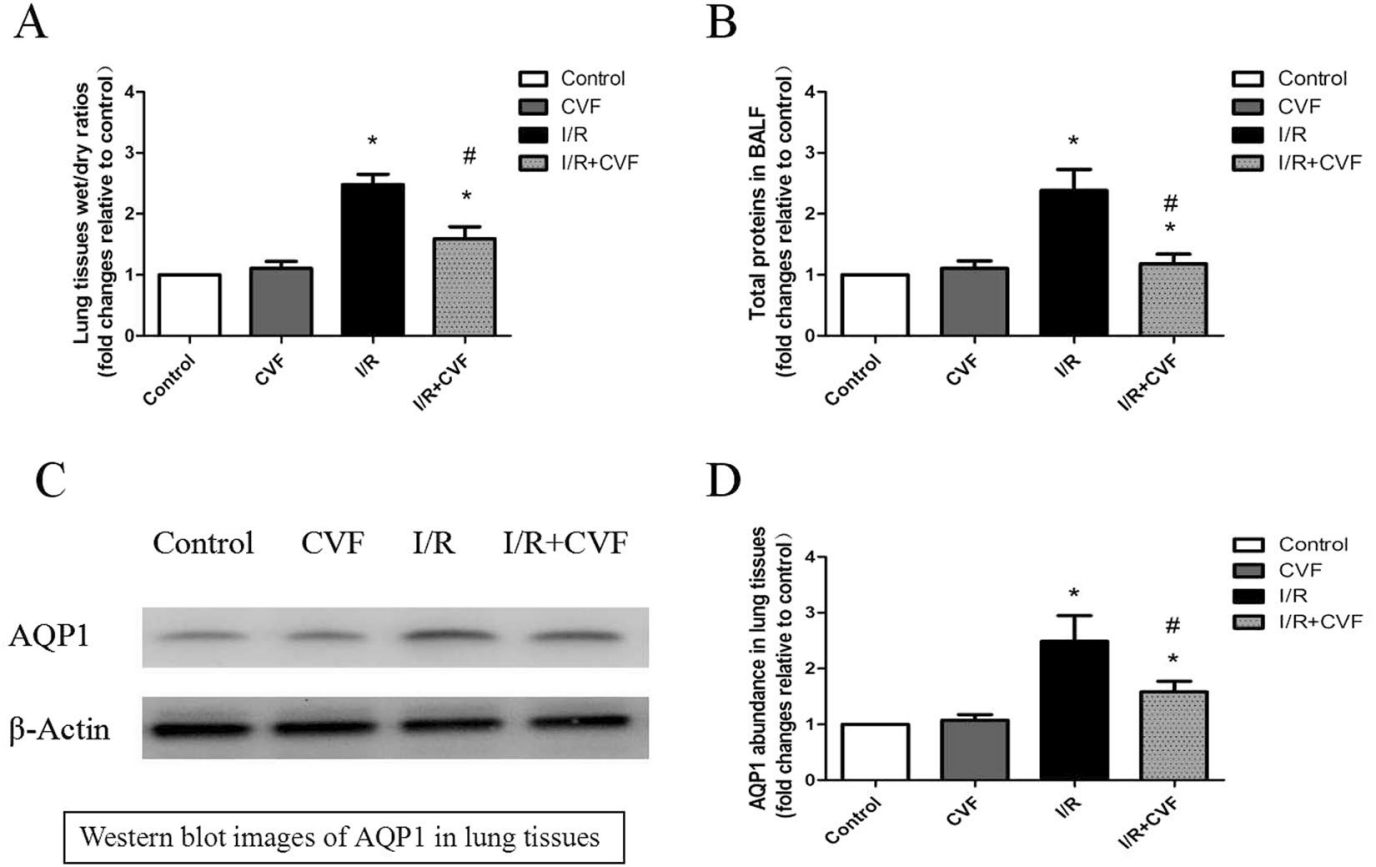
CVF pretreatment decreases I/R-induced pulmonary permeability and edema. (**A**) Graphic presentation of the Wet/dry ratios in lung tissues. (**B**) Graphic presentation of total proteins in BALF. (**C**) Representative Western blot images of AQP1 in lung tissues. (**D**) Graphic presentation of AQP1 abundance in lung tissues (The grouping of gels/blots cropped from different parts of the same gel). (^*^P < 0.05, compared with control. ^#^*P* < 0.05, compared between I/R and I/R + CVF group).

Pulmonary wet/dry ratios, the total proteins in BALF, and the expression of AQP1 in lung tissues were measured to evaluate pulmonary permeability and edema. The wet/dry ratios in lung tissues were showed in Fig. 4A, the levels of total proteins in BALF were showed in Fig. 4B–D presented the expression of AQP1 in lung tissues. Compared with the control group, lung I/R significantly increased the wet/dry ratios, the expression of total proteins in BALF and AQP1 in lung tissues (*P* < 0.05). Compared with the I/R group, pretreatment with CVF significantly reduced the wet/dry ratios, the total proteins in BALF and the AQP1 expression in lung tissues (*P* < 0.05).

### Function of CVF in preserving integrity of tight junctions and blood-air barrier in IR condition

**Figure 5:**
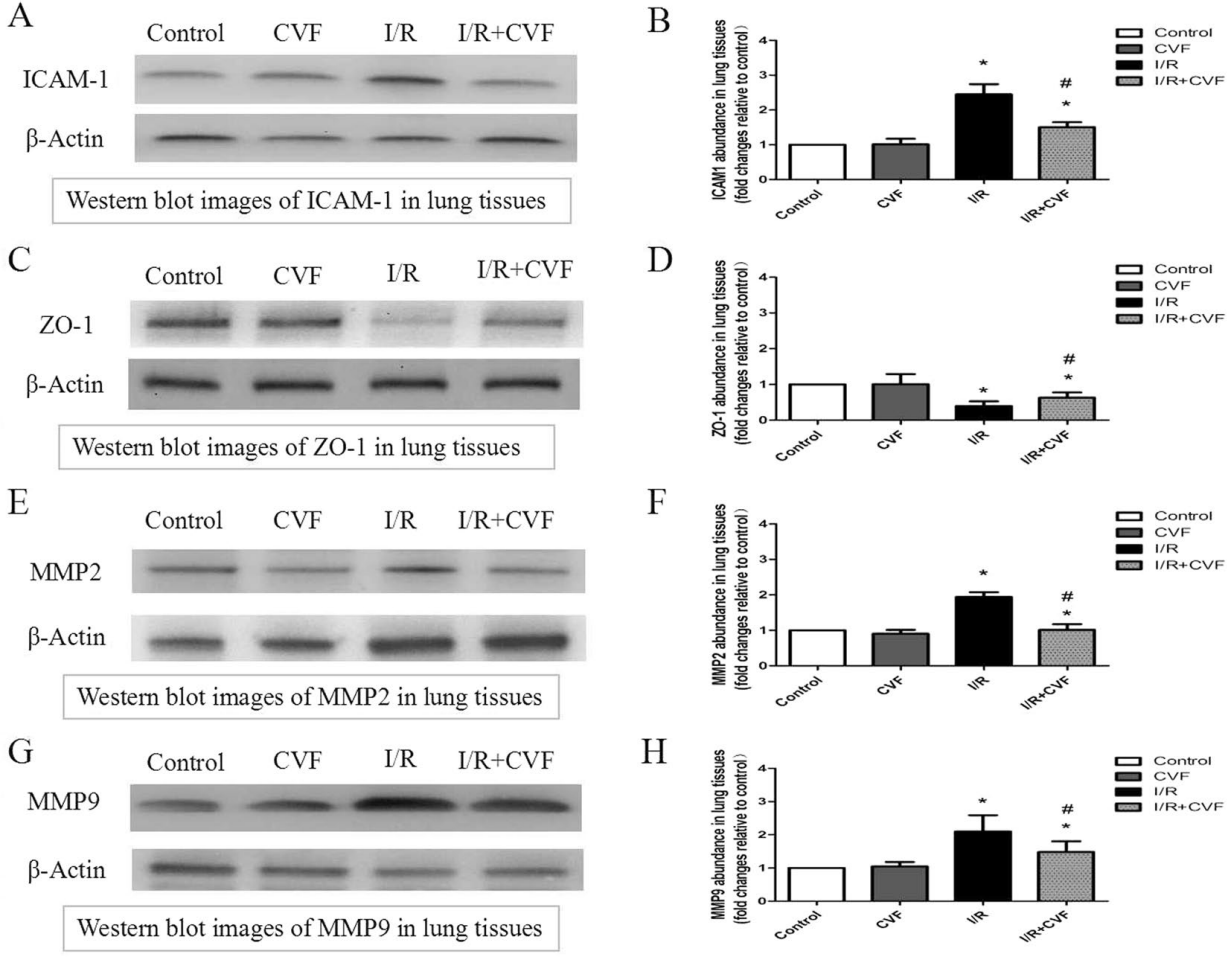
CVF pretreatment preserves integrity of tight junctions and blood-air barrier in IR condition. (**A**) Representative Western blot images of ICAM1 in lung tissues (The grouping of gels/blots cropped from different parts of the same gel). (**B**) Graphic presentation of ICAM1 abundance in lung tissues. (**C**) Representative Western blot images of ZO-1 in lung tissues (The grouping of gels/blots cropped from different parts of the same gel). (**D**) Graphic presentation of ZO-1 abundance in lung tissues. (**E**) Representative Western blot images of MMP2 in lung tissues (The grouping of gels/blots cropped from different parts of the same gel). (**F**) Graphic presentation of MMP2 abundance in lung tissues. (**G**) Representative Western blot images of MMP9 in lung tissues (The grouping of gels/blots cropped from different parts of the same gel). (**H**) Graphic presentation of MMP9 abundance in lung tissues. (^*^P < 0.05, compared with control. ^#^*P* < 0.05, compared between I/R and I/R + CVF group).

The protein expression of ICAM-1, ZO-1, MMP2, MMP9 in Lung tissues were measured by Western Blot Analysis to assess the integrity of tight junctions and blood-air barrier. The results in Fig. 5A,B showed that compared with the control group, the expression of ICAM-1 in lung tissues was significantly increased after lung I/R (*P* < 0.05). CVF pretreatment significantly inhibited the ICAM-1 expression induced by lung I/R (*P* < 0.05). The results in Fig. 5C,D demonstrated that compared with the control group, lung I/R had significantly reduced the expression of ZO-1 in lung tissues, and CVF pretreatment significantly increased ZO-1 expression compared with the I/R group (*P* < 0.05). Figure 5E–H showed that the expression of MMP2 and MMP9 in lung tissues significantly increased in I/R group compared with the control group (*P* < 0.05). CVF pretreatment significantly inhibited the I/R-induced MMP2 and MMP9 expression (*P* < 0.05). The results demonstrated that pretreatment with CVF had the function of preserving integrity of tight junctions and blood-air barrier in I/R condition.

## Discussion

During the occurring and developing progressions of the diseases, complement system is activated producing some pivotal complement components such as C3 and C5^21–23^. More and more studies have indicated that the excessive complement activation is intimately related to the initiation and amplification of the organs and tissues injury caused by I/R^24–27^. Consistent with the previous reports, our study showed that the levels of CH50 in plasma and C3 in plasma and BALF significantly enhanced after lung I/R. CVF is a structural and functional analogue of complement component C3^13^, which can cause continuously and excessively activation of C3 resulting in complement components depletion in serum. In this study, pretreatment with CVF significantly reduced the expression of complement component in plasma and BALF, which demonstrated complement depletion induced by CVF in a rat model.

The complement system plays a pivotal role in activating the innate immunity. However, overwhelming activation of complement has a close relationship with excessive immune activation and inflammatory eruption resulting in severe tissue injury. Some previous studies showed that the process of I/R initiated a complement activation and inflammatory response, which accompanied with releasing complement component and pro-inflammatory factors, attracting inflammatory cells and lysosomal enzymes, increasing small vasodilatation and pulmonary vascular permeability^8,28^. In this study, we found that after lung I/R, lung injury was obvious in morphology, the alveolar septums were ruptured, lung tissue structures were ambiguous, a large number of red blood cells and inflammatory cells infiltration were observed. Pretreatment with CVF could significantly ameliorate the damage of lung tissues induced by lung I/R. This effects of CVF demonstrated that complement activity was involved in the pathogenesis of LIRI. This study also showed that CVF significantly inhibited the IL-1β, IL-6, TNF-α and MPO expression in lung tissues and BALF in lung I/R condition. MPO is the marker lysosomal enzyme of polymorphonuclear cells (PMN), and MPO activity in lung tissues and BALF reflects the extent of PMN infiltration. Our results revealed that complement depletion induced by CVF pretreatment could reduce the pro-inflammatory factors release and PMN infiltration, which was contributed to relieve the damage of lung tissues in the process of lung I/R. The results are consistent with some previous studies. Hu *et al*.^29^ found that autophagy mediated by C5aR could induced alveolar macrophages apoptosis, disrupted pulmonary homeostasis and contributed to the development of ALI in a intestinal I/R mice model. Mao *et al*.^17^ have suggested that CVF could significantly reduce acute lung tissues damage caused by I/R, the mechanism might be inhibition of oxidant generation, infiltration of neutrophils. Proctor *et al*.^30^ found that complement inhibitors could selectively attenuate tissues injury following administration of CVF to rats. But some studies have the opposite conclusion. Glasgow *et al*.^31^ found that complement depletion induced by preoperative administration of CVF resulted in increased pulmonary inflammation following liver cryo injury via relative upregulation of NF-kappaB activity.

To assess pulmonary permeability and edema, we had measured pulmonary wet/dry ratios, the expression of total proteins in BALF and AQP1 in lung tissues. Our results were as anticipated, that CVF pretreatment significantly reduced the wet/dry ratios, the levels of total proteins in BALF and AQP1 expression after lung IR. This result was consistent with the result of the pathological morphology.

Furthermore, to assess the integrity of blood-air barrier, we evaluated the effect of CVF pretreatment on the ICAM-1, ZO-1, MMP2, MMP9 protein expression in Lung tissues. ICAM-1 is an endothelial-associated transmembrane protein, and has an important role in stabilizing cell-cell interactions. ICAM-1 expression can significantly increase caused by tissues injury^32^. ZO-1 is one of the essential members in composing intact cell tight junctions which is vital to maintain the integrity of microvascular barrier^33^. The main function of MMP2 and MMP9 is to degrade and restore the extracellular matrix and maintain the dynamic balance in basement membrane. MMP2 and MMP9 can destroy the basement membrane and promote inflammation, play an important role in reconstruction extracellular matrix in the process of tissues injury^34^. In this study, ICAM-1, MMP2, MMP9 significantly increased and ZO-1 significantly reduced in lung I/R rat animals, pretreatment with CVF can change the expressions of ICAM-1, ZO-1, MMP2, MMP9. The results suggested that lung I/R can lead to severe damage of blood-air barrier, and CVF pretreatment had the function of preserving integrity of tight junctions and blood-air barrier in I/R condition.

In this study, our data indicated illuminated that complement activation play an important role in the pathological process of LIRI, complement depletion induced by CVF pretreatment might be an option to prevent and cure tissue injury caused by I/R. However, Some conditions still restrict the use of CVF in clinical practice. For example, complement depletion by CVF can decrease the immune function, CVF affects all organs of the body and cannot aim at the injured organ. More innovative studies are needed if we want to realize the use of CVF in clinical condition.

## Conclusion

We come to the conclusion that CVF pretreatment could ameliorate LIRI and inhibit I/R-induced inflammatory response. The mechanisms of its protective effects might be alleviated blood-air barrier damage.
